# Three Circulating miRNAs Related to Non-Small-Cell Lung Cancer Progression: An Integrative Analysis of Their Biological Roles

**DOI:** 10.3390/biology14040399

**Published:** 2025-04-10

**Authors:** Yanqin Niu, Gaohui Fu, Sijian Xia, Menglong Li, Lin Qiu, Jun Wang, Kang Kang, Deming Gou

**Affiliations:** 1 Shenzhen Key Laboratory of Microbial Genetic Engineering, Vascular Disease Research Center, College of Life Sciences and Oceanography, Guangdong Provincial Key Laboratory of Regional Immunity and Disease, Carson International Cancer Center, School of Medicine, Shenzhen University, Shenzhen 518060, China; niuyq@szu.edu.cn (Y.N.);; 2 Department of Biochemistry and Molecular Biology, Shenzhen University Medical School, Shenzhen 518060, China

**Keywords:** biomarker discovery, microRNA, NSCLC (non-small-cell lung cancer), bioinformatics analysis

## Abstract

MicroRNAs (miRNAs) are crucial in physiological and pathological processes and serve as biomarkers for various diseases. We previously validated seven miRNA biomarkers and nine in lung adenocarcinoma (LUAD) and lung squamous cell carcinoma (LUSC). In this study, we observed distinct clustering patterns of LUAD or LUSC tissues compared to paired normal tissues based on miRNA expression levels, suggesting the potential involvement of circulating miRNAs in non-small-cell lung cancer (NSCLC) progression. To elucidate their biological function, we identified the most significant differentially expressed miRNAs (DE-miRNAs)—hsa-miR-451a, hsa-miR-139-5p and hsa-miR-126-5p—using The Cancer Genome Atlas (TCGA) and Gene Expression Omnibus (GEO) datasets. We then performed protein–protein interaction (PPI) analysis and constructed a miRNA-hub gene regulatory network based on targets predicted by several miRNA-target prediction tools. Additionally, we evaluated the biological functions of these miRNA biomarkers through EdU and wound healing assays in A549 cells. Our study identifies three miRNAs that may contribute to lung cancer progression by modulating cancer-related targets and highlights their potential as biomarkers. Future mechanistic investigations may provide novel insights into NSCLC pathogenesis and open new therapeutic avenues.

## Simple Summary

Lung cancer, especially non-small-cell lung cancer (NSCLC), is one of the leading causes of cancer-related deaths worldwide. In this study, we explored three microRNAs (miRNAs) found in the blood that could potentially serve as biomarkers for monitoring the progression of lung cancer. Our analysis revealed that these miRNAs were significantly altered in cancerous lung tissues compared to normal tissues. Specifically, we identified hsa-miR-451a, hsa-miR-139-5p, and hsa-miR-126-5p as key players in the development of lung cancer, which may aid in its diagnosis and prognosis. These findings could enhance early detection and provide new therapeutic insights for NSCLC, ultimately improving patient care and public health outcomes.

## 1. Introduction

Lung cancer is a leading cause of cancer-related mortality worldwide, comprising small-cell lung carcinoma (SCLC) and non-small-cell lung cancer (NSCLC), the latter being the predominant form accounting for 85% of cases, including lung adenocarcinoma (LUAD) and lung squamous cell carcinoma (LUSC) [1,2]. Despite advancements in treatments, the 5-year survival rates for lung cancer remain low, emphasizing the need for novel therapeutic targets [3].

MicroRNAs (miRNAs), small noncoding RNA molecules, regulate gene expression post-transcriptionally and are implicated in various diseases, including lung cancer [4,5,6,7]. In 2011, Hanahan et al. outlined eight hallmark capabilities of cancer, encompassing aspects such as sustaining proliferative signaling, evading growth suppressors, activating invasion and metastasis, enabling replicative immortality, inducing angiogenesis, resisting cell death, avoiding immune destruction, and perturbing cellular energetics [8]. miRNAs are inevitably intertwined with each of these hallmark capabilities [9]. For instance, miR-134 directly targets EGFR, inhibiting NSCLC cell proliferation by inducing cell cycle arrest and apoptosis [10]; the miR-29 family (comprising 29a, 29b, and 29c) intricately complement the 3′-UTRs of DNA methyltransferase (DNMT) 3A and 3B, critical enzymes involved in DNA methylation that are often upregulated in lung cancer and associated with poor prognosis [11]; epithelial-to-mesenchymal transition (EMT), characterized by the loss of E-cadherin-mediated cell adhesion and increased cell motility, tumor invasion, and metastasis, is regulated by miR-200, functioning as a cell-autonomous suppressor of EMT and metastasis by targeting PD-L1 [12]. These findings underscore the close connection between miRNAs and the progression of lung cancer, suggesting their significance as regulators in lung cancer pathogenesis.

miRNAs, initially studied in tissues or cells, have shown promise as noninvasive biomarkers for cancer risk assessment [13,14,15]. Circulating miRNAs offer insights into tumor classification, staging, and metastasis [16,17,18,19]. Some studies suggest a functional role of tumor-secreted circulating miRNAs in cancer behavior [20,21,22]. We previously validated seven potential circulating biomarkers for LUAD and nine for LUSC [23]. In this study, we observed distinct clustering patterns in NSCLC based on miRNA expression in The Cancer Genome Atlas (TCGA).

Furthermore, we analyzed the clinical significance of the most significant differentially expressed miRNAs (DE-miRNAs), conducted Gene Ontology (GO) and Kyoto Encyclopedia of Genes and Genomes (KEGG) analyses of target genes, constructed protein–protein interaction (PPI) networks to identify hub genes, and elucidated potential regulatory mechanisms in NSCLC. Additionally, we validated the biological functions of 11 miRNAs in A549 cells through proliferation and migration assays. The primary objective of this study is to reaffirm the identity of miRNA biomarkers in NSCLC tissues, offering fresh insights into their mechanisms.

## 2. Materials and Methods

### 2.1. miRNAs Microarray Data

We gathered miRNA sequencing data from 45 LUAD and 44 LUSC tissues, along with paired normal lung samples obtained from TCGA: “http://cancergenome.nih.gov/” (accessed on 1 January 2025). Additionally, we retrieved miRNA microarray data from the GEO database: “http://www.ncbi.nlm.nih.gov/geo” (accessed on 1 January 2025) by employing specific search criteria. These criteria encompassed “non-small-cell lung cancer miRNA” as the study keyword, “noncoding RNA profiles by array” as the study type, “Homo sapiens” as the organism, and “tissues” as the attribute name and were aimed at identifying NSCLC-associated miRNA datasets.

Subsequently, we conducted a detailed analysis of the GSE53882, GSE36681, and GSE15008 datasets, which included data derived from matched frozen tissues comprising cancer tissues and adjacent normal tissues. The GSE53882 dataset, generated via the GPL18130 platform, comprised data from 397 NSCLC patients and 151 corresponding adjacent noncancerous tissues. The GSE36681 dataset provided miRNA expression profiles for 56 pairs of lung adenocarcinoma samples. Lastly, the GSE15008 dataset, utilizing the GPL8176 platform, encompassed 116 pairs of primary tumor tissues and their corresponding adjacent normal tissues. To facilitate our analysis, we downloaded all miRNA expression matrices and probe tables and subsequently merged them using the GEO query R package [24].

### 2.2. Survival Analysis

We conducted survival analysis in NSCLC patients using the Kaplan–Meier plotter: “http://kmplot.com/analysis/index.php?p=service” (accessed on 1 January 2025). For this analysis, survival curves were generated based on high and low expression levels, and patients were categorized by employing the “Auto select best cutoff” method.

### 2.3. Prediction of miRNA Target Genes

Target genes were predicted using four online tools: DIANA: “http://carolina.imis.athena-innovation.gr/diana_tools/web/index.php?r=tarbasev8/index” (accessed on 1 January 2025), miRDB: “http://mirdb.org/” (accessed on 1 January 2025), miRTarBase: “http://mirtoolsgallery.tech/mirtoolsgallery/node/1080” (accessed on 1 January 2025), and TargetScan: “http://www.targetscan.org/vert_72/” (accessed on 1 January 2025). A Venn diagram was generated with the help of the online tool jvenn: “http://jvenn.toulouse.inra.fr/app/example.html” (accessed on 1 January 2025), illustrating the common targets obtained from these four websites.

### 2.4. Expression and Function of Target Genes

GEPIA “http://gepia.cancer-pku.cn/” (accessed on 1 January 2025) offers rapid and customizable functions, encompassing differential expression analysis and patient survival analysis of miRNA targets, relying on data from TCGA and GTEx [25]. We retrieved the expression patterns of target genes from a total of 483 LUAD and 347 normal samples, as well as 486 LUSC and 338 normal samples, respectively [25]. To further annotate the functions of genes targeted by DE-miRNAs, the DAVID program: “https://david-d.ncifcrf.gov/” (accessed on 1 January 2025) was utilized to perform GO and KEGG pathway analyses. A *p* value < 0.05 was considered significant [26,27].

### 2.5. Cell Proliferation and Migration

We procured miRNA mimics from Suzhou GenePharma Co., Ltd. (Suzhou, China). A549 cells (purchased from The Global Bioresource Center, ATCC, Manassas, VA, USA) were seeded in triplicate in 24- or 48-well plates at an appropriate density and cultured for 24 h. Subsequently, the cells were transfected with miRNA/control mimic (40 nM) using TurboFect™ Transfection Reagent (Thermo Fisher Scientific, Waltham, MA, USA) for a duration of 72 h. Cell proliferation was assessed using the EdU Assay Kit (Ribobio, Guangzhou, China), as per the provided protocol. Images were captured and analyzed utilizing a Lionheart FX Automated Live Cell Imager (BioTek, Winooski, VT, USA), consistent with our previous publication methodology [28].

For the migration assay, we performed a wound healing assay following the methodology outlined in our previously published paper [28]. Specifically, cell-free gaps were generated using the BioTek Autoscratch (BioTek, Winooski, VT, USA) and then maintained in DMEM containing 0.2% FBS. Images were acquired and analyzed using the Lionheart FX Automated Live Cell Imager at both 0 h and 48 h. Migration rates were determined by measuring the reduction in the average distance of the lines as the wound closed.

### 2.6. Data Analysis

Analysis of miRNA expression levels in TCGA was performed utilizing the R packages TGGAbiolinks [29], while analysis of miRNA expression in each GEO dataset was carried out using the limma package [30]. We employed principal component analysis (PCA) to examine the global expression patterns of miRNAs in lung cancer tissues and paired normal tissues. This method allows for the visualization of clustering patterns and identification of underlying trends in the data, aiding in the differentiation between cancerous and normal tissues. PCA was carried out using the Factoextra R package: “https://cloud.r-project.org/package=factoextra/” (accessed on 1 January 2025). GO analysis was performed to classify the biological roles of the target genes of differentially expressed miRNAs. This method categorizes gene functions into three major areas: biological processes (BP), cellular components (CC), and molecular functions (MF). KEGG pathway enrichment analysis was used to explore the signaling pathways associated with the target genes regulated by the DE-miRNAs. KEGG analysis provided a comprehensive view of significantly enriched pathways, helping to identify key molecular mechanisms involved in lung cancer development and progression. Both GO and KEGG analyses were conducted using the ClusterProfiler R package: “https://www.bioconductor.org/packages/release/bioc/html/clusterProfiler.html” (accessed on 1 January 2025).

Comparisons of miRNA expressions in tissues were executed through a two-tailed Student’s *t*-test employing GraphPad Prism 8.0 (GraphPad Software, Inc., La Jolla, CA, USA). The data were presented as means ± SE (standard error), and variables with *p*-values less than 0.05 were considered statistically significant.

## 3. Results

### 3.1. Ten Circulating miRNAs Validated in Lung Cancer Tissues

**Figure 1:**
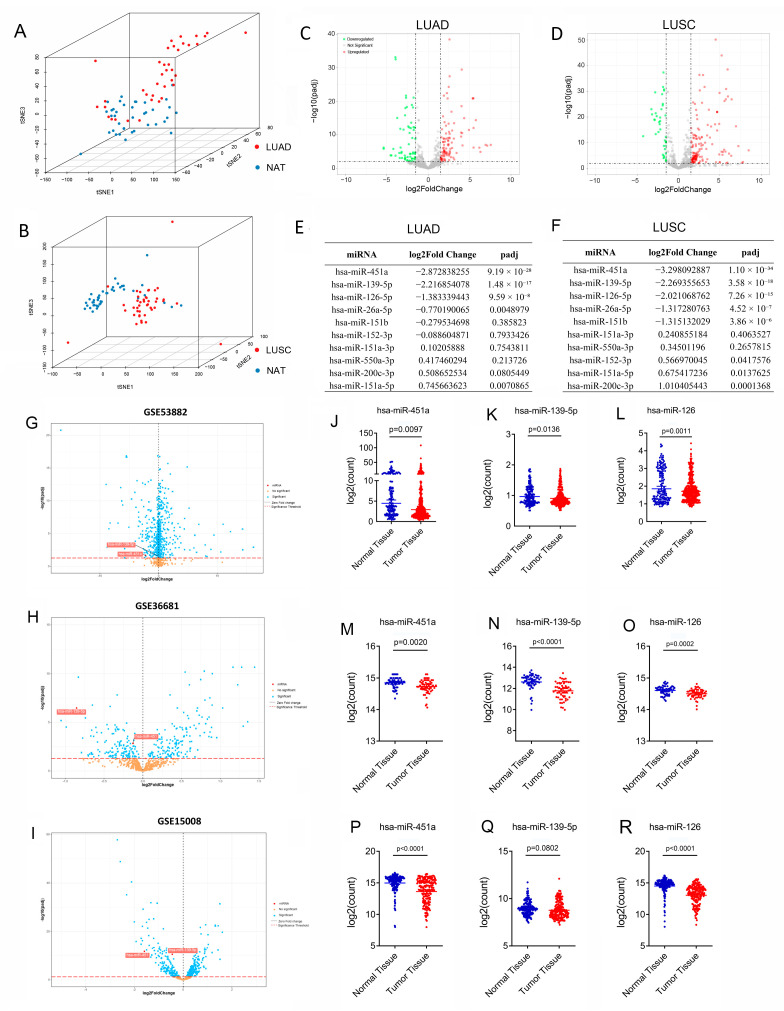
Expression levels of 10 potential miRNA biomarkers in non-small-cell lung cancer (NSCLC) tumors and adjacent normal tissues. (**A**) PCA graph showing multidimensional scaling of 45 pairs of LUAD tumors and adjacent normal tissues from the TCGA dataset based on 6 miRNAs (hsa-miR-26a-5p, hsa-miR-126-5p, hsa-miR-139-5p, hsa-miR-152-3p, hsa-miR-451a, and hsa-miR-200c-3p). (**B**) A total of 44 pairs of lung LUSC tumors and adjacent normal tissues from the TCGA dataset clustered separately based on the expression status of 8 miRNAs (hsa-miR-26a-5p, hsa-miR-126-5p, hsa-miR-139-5p, hsa-miR-151a-3p, hsa-miR-151a-5p, hsa-miR-151b, hsa-miR-152-3p, and hsa-miR-550a-3p). (**C**) Volcano plot illustrating the expression levels of 2213 miRNAs in 45 LUAD tumors and 45 adjacent normal tissues. (**D**) Profiling of 2219 miRNAs in 44 LUSC tumors and 44 adjacent normal tissues. (**E**,**F**) Fold change of 10 circulating miRNAs in LUAD and LUSC in TCGA. (**G**) Volcano plot showing 1888 miRNA expression profiles obtained from 397 patients with NSCLC and 151 corresponding adjacent normal tissues in GSE53882 dataset (frozen samples). (**H**) Profiling of 858 miRNAs in matched lung adenocarcinoma and uninvolved lung tissues using 56 pairs of fresh-frozen samples from never smokers in the GSE36681 dataset. (**I**) Profiling of 818 miRNas in 187 primary lung cancers and 174 corresponding adjacent normal lung tissues collected a minimum of 5 cm from the tumor in the GSE15008 dataset. (**J**–**L**) Expression levels of hsa-miR-451a, hsa-miR-139-5p and hsa-miR-126-5p in the GSE53882 dataset. (**M**–**O**) Expression levels of hsa-miR-451a, hsa-miR-139-5p and hsa-miR-126-5p in the GSE36681 dataset. (**P**–**R**) Expression levels of hsa-miR-451a, hsa-miR-139-5p and hsa-miR-126-5p in the GSE15008 dataset. Bar charts represent the mean ± SE, and Student’s *t*-test was used for statistical analysis.

In a previous study, we validated seven miRNAs (hsa-miR-26a-5p, hsa-miR-126-5p, hsa-miR-139-5p, hsa-miR-152-3p, hsa-miR-451a, hsa-miR-200c-3p, and hsa-miR-3135b) and nine miRNAs (hsa-miR-26a-5p, hsa-miR-126-5p, hsa-miR-139-5p, hsa-miR-151a-3p, hsa-miR-151a-5p, hsa-miR-151b, hsa-miR-152-3p, hsa-miR-550a-3p, and hsa-miR-3135b) as potential biomarkers for LUAD and LUSC from a panel of 486 cancer-related miRNAs [23]. To validate the relevance of these circulating miRNAs in tissues, we obtained miRNA sequencing data for 10 miRNAs (with information for hsa-miR-3135b being unavailable) from 45 LUAD and 44 LUSC tissues, along with matched normal lung samples from TCGA. Principal component analysis (PCA) revealed that LUAD and LUSC tissues clustered differently from paired normal tissues based on the expression levels of six and eight miRNAs, respectively (Figure 1A,B). We compared the expression levels of these miRNAs in 45 pairs of LUAD samples (Figure 1C, Table S1) and 44 pairs of LUSC tissue samples (Figure 1D, Table S2) and found that hsa-miR-451a, hsa-miR-139-5p, and hsa-miR-126-5p displayed the most significant differences in both LUAD and LUSC (Figure 1E,F). Finally, we revalidated the expression levels of hsa-miR-451a, hsa-miR-139-5p, and hsa-miR-126-5p in three miRNA microarray datasets (GSE53882, GSE36681, and GSE15008), and these three miRNAs were significantly decreased in lung cancer tissues compared with normal tissues, except for hsa-miR-139-5p, which was only slightly reduced in the GSE15008 dataset without significance (Figure 1G–R, Tables S3–S5).

### 3.2. Confirmation of Three DE-miRNAs’ Clinical Significance

**Figure 2:**
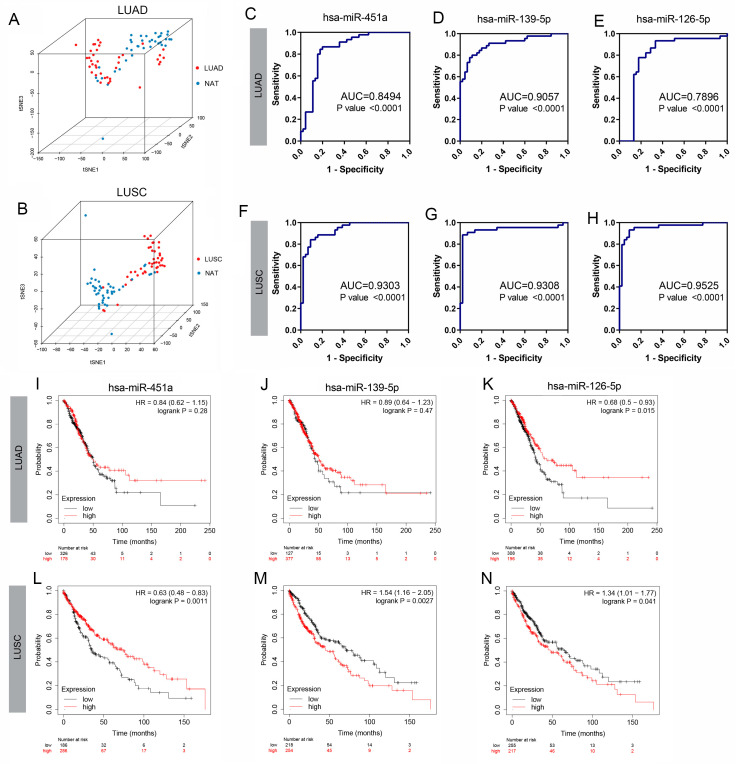
Potential clinical value analysis of three DE-miRNAs in NSCLC. (**A**) PCA graph showing multidimensional scaling of 45 pairs of LUAD tumors and adjacent normal tissues enrolled in the TCGA dataset based on the expression of 3 miRNAs (hsa-miR-26a-5p, hsa-miR-126-5p, and hsa-miR-451a). (**B**) PCA graph showing multidimensional scaling of 44 pairs of LUSC tumors and adjacent normal tissues from the TCGA dataset clustered separately based on the expression status of the 3 miRNAs (hsa-miR-26a-5p, hsa-miR-126-5p, and hsa-miR-451a). (**C**–**E**) ROC of hsa-miR-451a, hsa-miR-139-5p and hsa-miR-126-5p in LUAD. (**F**–**H**) ROC of hsa-miR-451a, hsa-miR-139-5p and hsa-miR-126-5p in LUSC. (**I**–**K**) Kaplan–Meier survival curves of hsa-miR-451a, hsa-miR-139-5p and hsa-miR-126-5p in LUAD. (**L**–**N**) Kaplan–Meier survival curves of hsa-miR-451a, hsa-miR-139-5p and hsa-miR-126-5p in LUSC.

To evaluate the clinical significance of the most significantly different miRNAs (hsa-miR-451a, hsa-miR-139-5p, and hsa-miR-126-5p), we further analyzed the expression levels obtained from 45 LUAD and 44 LUSC tissues, as well as those from paired normal lung samples from the TCGA database. The PCA graph revealed that lung cancer tissues and normal tissues clustered differently based solely on the expression levels of hsa-miR-451a, hsa-miR-139-5p, and hsa-miR-126-5p (Figure 2A,B).

Additionally, ROC analysis based on the expression level of each individual miRNA was conducted. The area under the ROC curve (AUC) for hsa-miR-451a, hsa-miR-139-5p, and hsa-miR-126-5p was 0.8494, 0.9057, and 0.7896 (*p*-value < 0.0001) in LUAD, respectively. The corresponding values for LUSC were 0.9303, 0.9308, and 0.9525 (*p*-value < 0.0001), suggesting the potential utility of these three miRNAs in distinguishing lung cancer from normal tissues (Figure 2C–H).

Furthermore, the probability of survival was assessed using the Kaplan–Meier plotter based on miRNA expression levels. High expression levels of hsa-miR-451a were associated with a better prognosis for patients with LUSC (Figure 2L). In contrast, high expression levels of hsa-miR-139-5p indicated a poor clinical outcome in LUSC (Figure 2M). There were no significant differences in hsa-miR-451a and hsa-miR-139-5p expression levels between the high and low expression groups in LUAD (Figure 2I,J). As for hsa-miR-126-5p, higher levels were indicative of a higher probability of survival in LUAD (Figure 2K) but a lower probability in LUSC (Figure 2N).

### 3.3. Function and Pathway Analysis of Predicted Target Genes

**Figure 3:**
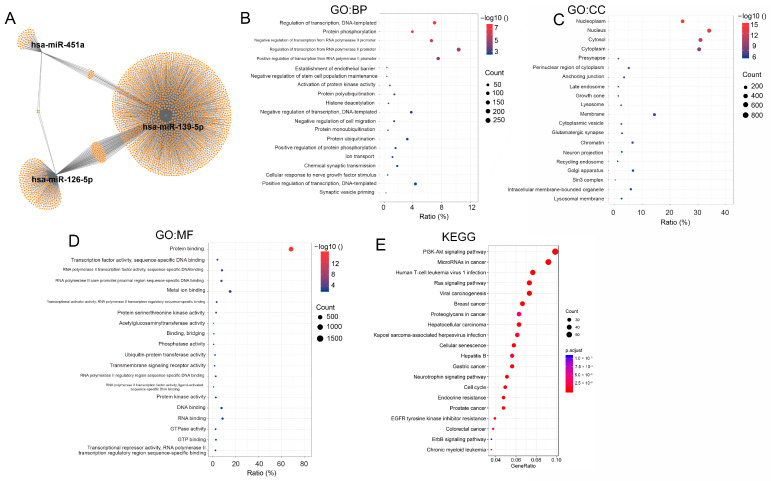
GO and KEGG enrichment analyses of potential targets for three DE-miRNAs. (**A**) The miRNA-target gene network for hsa-miR-451a, hsa-miR-139-5p, and hsa-miR-126-5p was constructed using the online tool miRWalk. (**B**–**D**) Enriched biological processes (BP), cellular components (CC), and molecular functions (MF) of target genes for hsa-miR-451a, hsa-miR-139-5p, and hsa-miR-126-5p in the Gene Ontology analysis. (**E**) KEGG pathway enrichment analysis of potential target genes for hsa-miR-451a, hsa-miR-139-5p, and hsa-miR-126-5p.

To evaluate the biological roles of the three DE-miRNAs in NSCLC, all potential target genes (Figure 3A) of these miRNAs, obtained from miRwalk, were used for GO function annotation and KEGG pathway enrichment. The top 20 GO terms, including biological process (BP), cellular component (CC), and molecular function (MF), are depicted in Figure 3B–D. In the BP analysis, these genes were significantly enriched in “Regulation of transcription from RNA polymerase II promoter”, “Positive regulation of transcription from RNA polymerase II promoter”, and “Regulation of transcription, DNA-templated”. In the CC analysis, these genes were mainly enriched in “Nucleus”, “Cytosol”, and “Cytoplasm”. The MF analysis showed that the target genes were particularly enriched in “Protein binding”, “Metal ion binding”, and “RNA binding”. KEGG pathway analysis revealed significant enrichment of these target genes in “PI3K-Akt signaling pathway”, “microRNAs in cancer”, and “Human T-cell leukemia virus 1 infection” (Figure 3E).

### 3.4. Potential Clinical Value of Predicted Target Genes of DE-miRNAs

**Figure 4:**
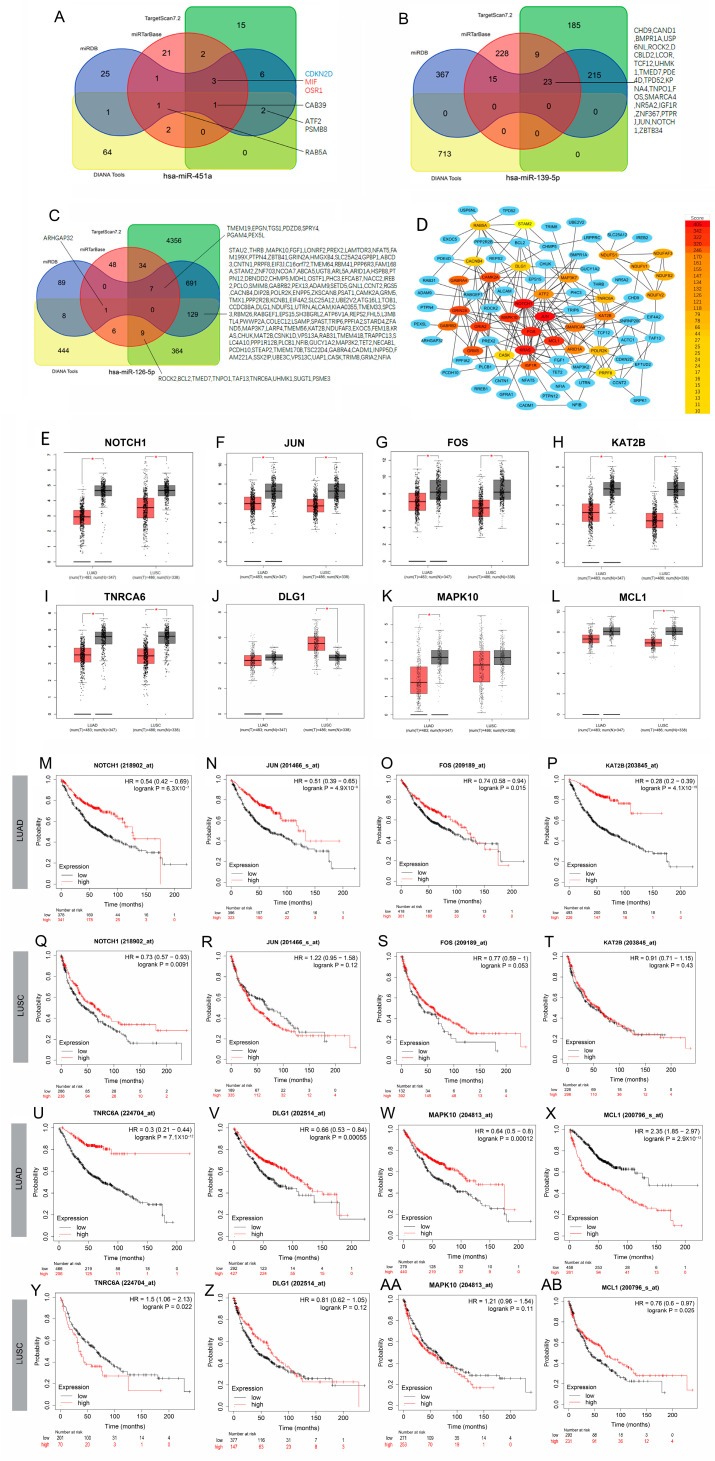
The expression levels, interactions, and potential clinical value of potential target genes of three DE-miRNAs. (**A**–**C**) Venn diagram showing common targets of hsa-miR-451a, hsa-miR-139-5p and hsa-miR-126-5p obtained from four miRNA-target prediction websites, DIANA, miRDB, miRTarBase and TargetScan. (**D**) The PPI network of potential target genes of three DE-miRNAs. (**E**–**L**) The expression levels of 8 hub genes in LUAD or LUSC based on the TCGA and GTEx datasets. The asterisk (*) represents a statistically significant difference between tumor and normal tissues, based on the GEPIA platform’s default *p*-value cutoff of 0.01. (**M**–**AB**) The prognostic analysis of 8 hub genes in LUAD or LUSC.

To explore the biological role of the three DE-miRNAs in NSCLC, we obtained potential target genes from four online tools: DIANA, miRDB, miRTarBase, and TargetScan. A total of 172 genes were identified, including seven targets of hsa-miR-451a, 23 targets of hsa-miR-139-5p, and 146 targets of hsa-miR-126-5p, all of which were validated by more than three tools (Figure 4A–C). Subsequently, we constructed a protein–protein interaction (PPI) network using the STRING database and identified hub genes. These hub genes were visualized using Cytoscape software (Version:3.10.3) (Figure 4D).

We further analyzed the expression levels of 28 hub genes (with a score > 10) and their impact on patient survival using the online tool GEPIA and the Kaplan–Meier plotter. The results for NOTCH1, JUN, FOS, KAT2B, TNRC6A, DLG1, MAPK10, and MCL1 were presented as they showed better potential in distinguishing normal tissues from LUAD or LUSC tissues (Figure 4E–L).

As depicted in Figure 4M–AB, higher expression levels of NOTCH1, JUN, FOS, KAT2B, TNRC6A, DLG1, and MAPK10 were associated with a better prognosis for patients with LUAD. Conversely, higher expression levels of MCL1 indicated a lower probability of survival in LUAD. In the case of LUSC, there were significant differences in the prognosis of NOTCH1, TNRC6A, and MCL1 between the high and low expression groups, while no significant differences were observed in the case of JUN, FOS, KAT2B, DLG1, and MAPK10. Specifically, higher expression levels of NOTCH1 and MCL1 indicated a higher probability of survival, while a higher expression level of TNRC6A indicated a lower probability of survival in LUSC.

### 3.5. Functions of 11 miRNAs in A549 Cells

**Figure 5:**
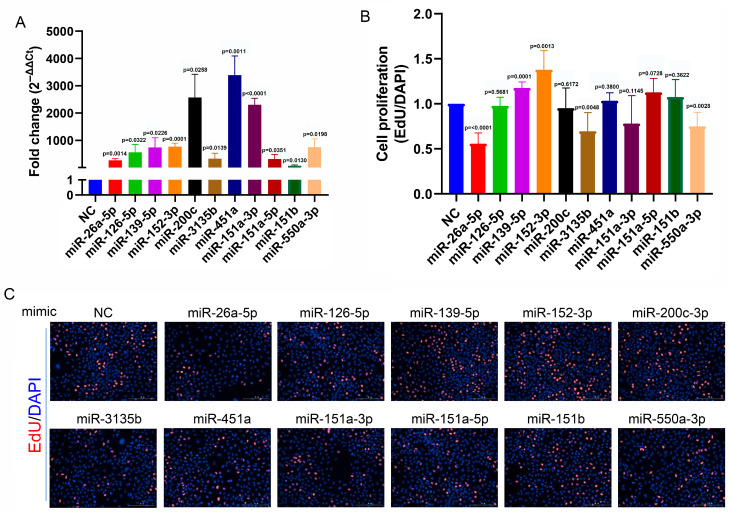
miRNA overexpression interferes with the proliferation of A549 cells. (**A**) A549 cells were transfected with miRNA mimics to overexpress miRNAs, and the miRNA expression levels were quantified by RT-qPCR. (**B**) Proliferation rates of A549 cells were assessed using the EdU incorporation assay. (**C**) Representative images of the EdU assay comparing miRNA mimics and control mimics. Bar charts represent the mean ± standard deviation, and statistical analysis was performed using Student’s *t*-test. All the tests were repeated 3~4 times.

**Figure 6:**
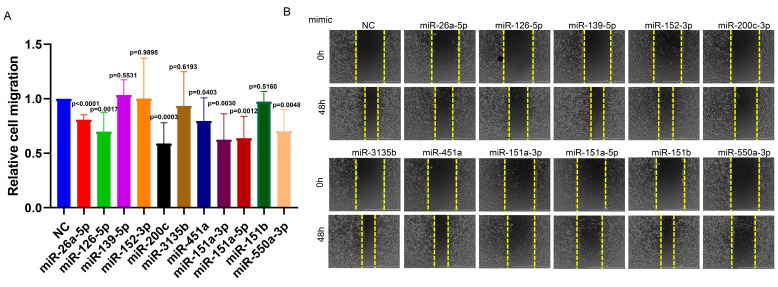
miRNA overexpression affects the migration of A549 cells. (**A**) The migration rate was assessed using a wound healing assay. (**B**) Representative images showing the average distance between wound edges at 0 h and 48 h. Bar charts represent the mean ± standard deviation, and statistical analysis was conducted using Student’s *t*-test. All experiments were repeated 3–4 times.

Abnormal proliferation and migration of NSCLC cells are critical factors in tumor progression. To investigate whether these 11 circulating miRNA biomarkers play a role in the molecular events underlying tumor progression, we overexpressed miRNAs by transfecting miRNA mimics into A549 cells (Figure 5A). As illustrated in Figure 5B,C, the overexpression of miR-26a, miR-3135b, and miR-550a-3p reduced the proliferation of A549 cells, while the overexpression of miR-139-5p and miR-152-3p increased cellular proliferation (Figure 5B,C). Moreover, the overexpression of miR-26a-5p, miR-126-5p, miR-200c, miR-451a, miR-151a-3p, miR-151a-5p, and miR-550-3p significantly suppressed the migration of A549 cells (Figure 6A,B).

## 4. Discussion

miRNAs have gained recognition as key players in physiological and pathological processes, with significant potential as biomarkers for various diseases due to their stability in bodily fluids over time. In our previous work, we identified 11 miRNA biomarkers with prognostic value in LUAD and LUSC [23]. However, the precise roles of these miRNAs in the transition from healthy lung function to NSCLC remain unclear, especially in longitudinal studies.

Despite the growing interest in miRNAs as potential biomarkers, much remains to be learned about their biological functions. In our study, we employed a PCA to assess the expression patterns of 10 out of the 11 miRNA biomarkers and found that normal lung tissues and NSCLC tissues clustered differently. This observation suggests a potential physiological relevance between circulating miRNA biomarkers and the pathogenesis and progression of NSCLC. Significantly, the three most differentially expressed miRNAs, namely, hsa-miR-451a, hsa-miR-139-5p, and hsa-miR-126-5p, were able to clearly distinguish between normal and NSCLC tissues.

Furthermore, these three miRNAs were consistently downregulated in both LUAD and LUSC tissues from the TCGA dataset and in NSCLC tissues from GEO datasets (GSE53882, GSE36681, and GSE15008). Additionally, they exhibited high diagnostic accuracy in distinguishing NSCLC patients from controls. Moreover, the expression levels of these miRNAs were significantly associated with overall survival in LUAD and LUSC. These findings collectively suggest that these three miRNAs may serve as predictive biomarkers and key regulators of the progression of LUAD and LUSC.

Our previous research had already demonstrated that hsa-miR-451a was downregulated in LUAD plasma, while both hsa-miR-139-5p and hsa-miR-126-5p were upregulated in LUAD and LUSC plasma [23]. Remarkably, our analysis of the TCGA database confirmed that these three miRNAs were significantly downregulated in LUAD and LUSC tissues. This observation may be attributed to the early dysregulation of some tumor suppressors and oncomiRs in lung cancer tumorigenesis [31]. Furthermore, certain miRNAs are packaged in microvesicles or associated with RNA-binding proteins and can be released into circulation. These miRNAs may then enter recipient cells, participating in signaling events [20] or influencing distant sites from the primary tumor, possibly contributing to the formation of pre-metastatic niches [32].

Global miRNA dysregulation, coupled with altered expression of their target mRNA transcripts, has emerged as a hallmark of cancer [33,34]. To gain insight into the functional pathways influenced by these three DE-miRNAs, we analyzed their target genes and focused on the pathways associated with these targets. Our results revealed that the potential target genes were enriched in pathways such as the PI3K-Akt signaling pathway, miRNAs in cancer, and human T-cell leukemia virus 1 infection, aligning with findings from previous studies [35].

To delve deeper into the roles of these three DE-miRNAs in cellular processes, we identified and characterized their target genes. After confirming the target genes via multiple miRNA-target prediction websites, we established a preliminary hub gene network, revealing the complexity of tumor development. The expression levels of several hub genes were significantly correlated with patient prognosis in LUAD and LUSC, underscoring their potential significance in disease progression.

The role of circulating miRNAs in disease biology has raised intriguing questions beyond their utility as biomarkers [36]. In our study, we provided evidence that three miRNAs not only have diagnostic and prognostic value but also play a role in the molecular events driving disease progression. These findings open new avenues for understanding the underlying molecular mechanisms of NSCLC. However, we acknowledge that our results present a preliminary hypothesis. Future research should focus on experimental validation of miRNA loading into extracellular vehicles (EVs), their secretory mechanisms, release into the bloodstream, incorporation into target cells, and their functional consequences. This will contribute to a more comprehensive understanding of the role of circulating miRNAs in disease biology. Moreover, although we correlated miRNA expression with patient survival and other clinical parameters, these associations were based on retrospective data analysis. Prospective studies with larger cohorts and comprehensive clinical data would strengthen the clinical relevance of miRNA biomarkers in NSCLC management.

## 5. Conclusions

In conclusion, our study has identified three circulating microRNAs (hsa-miR-451a, hsa-miR-139-5p, and hsa-miR-126-5p) as potential biomarkers for non-small-cell lung cancer (NSCLC). Through comprehensive analysis of publicly available datasets and experimental validation, we have demonstrated that these miRNAs are significantly downregulated in LUAD and LUSC tissues compared to their normal counterparts. The diagnostic and prognostic potential of these miRNAs is further supported by their ability to distinguish cancerous tissues from normal tissues, as well as their correlation with patient survival. Additionally, functional assays in A549 cells suggest that these miRNAs may influence key cancer-related pathways, contributing to tumor progression. Overall, our findings highlight the importance of these miRNAs in NSCLC pathogenesis and underscore their potential as non-invasive biomarkers for early diagnosis and therapeutic targeting. Further mechanistic studies are warranted to explore the underlying molecular mechanisms and validate the clinical applicability of these miRNAs in the management of NSCLC.

## Data Availability

The datasets analyzed for this study were obtained from TCGA dataset: “http://cancergenome.nih.gov/” (accessed on 1 January 2025) and GEO database: “http://www.ncbi.nlm.nih.gov/geo” (accessed on 1 January 2025).

## Supplementary Materials

The following supporting information can be downloaded at: https://www.mdpi.com/article/10.3390/biology14040399/s1, Table S1: Sequencing data of 10 miRNAs in 45 LUAD tumors and 45 adjacent normal tissues from Cancer Genome Atlas (TCGA). Table S2: Sequencing data of 10 miRNAs in 44 LUSC tumors and 44 adjacent normal tissues from Cancer Genome Atlas (TCGA). Table S3: Microarray data of 10 miRNAs obtained from 397 patients with NSCLC and 151 corresponding adjacent normal tissues in GSE53882 dataset. Table S4: Microarray data of 10 miRNAs obtained from matched lung adenocarcinoma and uninvolved lung using 56 pairs of fresh-frozen samples from never smokers in GSE36681 dataset. Table S5: Microarray data of 10 miRNAs obtained from 187 primary lung cancers and 174 corresponding adjacent normal lung tissues collected a minimum of 5 cm from the tumor in GSE15008 dataset.

## Abbreviations

The following abbreviations are used in this manuscript:

miRNA	microRNA
NSCLC	non-small-cell lung cancer
LUAD	lung adenocarcinoma
LUSC	lung squamous cell carcinoma
DE-miRNAs	differentially expressed miRNAs
ROC	receiver operating characteristic curve
GO	Gene Ontology
BP	biological process
CC	cellular component
MF	molecular function
KEGG	Kyoto Encyclopedia of Genes and Genomes
PPI	protein-protein interaction
TCGA	Cancer Genome Atlas
GEO	Gene Expression Omnibus
ROC	area under ROC curve
PCA	Principal component analysis
